# One bout of open skill exercise improves cross-modal perception and immediate memory in healthy older adults who habitually exercise

**DOI:** 10.1371/journal.pone.0178739

**Published:** 2017-06-01

**Authors:** Jessica O’Brien, Giovanni Ottoboni, Alessia Tessari, Annalisa Setti

**Affiliations:** 1 School of Applied Psychology, University College Cork, Cork, Ireland; 2 Department of Psychology, Bologna University, Bologna, Italy; University of Akron, UNITED STATES

## Abstract

One single bout of exercise can be associated with positive effects on cognition, due to physiological changes associated with muscular activity, increased arousal, and training of cognitive skills during exercise. While the positive effects of life-long physical activity on cognitive ageing are well demonstrated, it is not well established whether one bout of exercise is sufficient to register such benefits in older adults. The aim of this study was to test the effect of one bout of exercise on two cognitive processes essential to daily life and known to decline with ageing: audio-visual perception and immediate memory. Fifty-eight older adults took part in a quasi-experimental design study and were divided into three groups based on their habitual activity (open skill exercise (mean age = 69.65, SD = 5.64), closed skill exercise, N = 18, 94% female; sedentary activity-control group, N = 21, 62% female). They were then tested before and after their activity (duration between 60 and 80 minutes). Results showed improvement in sensitivity in audio-visual perception in the open skill group and improvements in one of the measures of immediate memory in both exercise groups, after controlling for baseline differences including global cognition and health. These findings indicate that immediate benefits for cross-modal perception and memory can be obtained after open skill exercise. However, improvements after closed skill exercise may be limited to memory benefits. Perceptual benefits are likely to be associated with arousal, while memory benefits may be due to the training effects provided by task requirements during exercise. The respective role of qualitative and quantitative differences between these activities in terms of immediate cognitive benefits should be further investigated. Importantly, the present results present the first evidence for a modulation of cross-modal perception by exercise, providing a plausible avenue for rehabilitation of cross-modal perception deficits, which are emerging as a significant contributor to functional decline in ageing.

## Introduction

There exists a well-documented link between physical exercise and cognition throughout the lifespan [1–3]. This association is particularly salient in the context of old age, when cognitive abilities naturally deteriorate [2,4]. The extant literature has predominately focused on chronic exercise studies, which explore the effects of long term exercise on cognition [5–7]. However, an emerging body of research is investigating the cognitive benefits from a single bout of exercise (also known as acute exercise) in isolation or in combination with exercise programmes [8,9].

The acute exercise literature is based on the premise that physiological changes induced by exercise impact cognition [10,11]. Increasing heart rate and changing plasma catecholamines, both associated with a heightened arousal, and the production of brain derived neurotropic factors, associated with hippocampal growth, are amongst the physiological changes discussed in the literature in relation to cognitive function [7]. While several meta-analyses report a small positive effect of acute exercise on cognitive functioning [10,12,13], a number of studies have found acute exercise had negligible effects on cognition or even impaired cognitive performance [14,15].

Inconsistent results may be due to moderator variables such as level of fatigue, duration of the exercise bout and exercise intensity [9,10,16]. One identified moderator is exercise mode. The modality of exercise seems to have differential effects on cognition [17,18] due to different cognitive demands and skills required. Exercise modality can be classified as either open skill or closed skill [18]. Open skill exercises are defined by a complex and an unpredictable environment and are cognitively demanding; examples include tennis and badminton [17]. In contrast, closed skill exercises such as swimming and running, involve a more predictable environment and require less cognitive effort [18].

Research to date suggests that open skill exercise may facilitate greater cognitive benefits due to higher cognitive investment during exercise [19], with dancing also showing promising results in older adults [1]. In terms of immediate effects of different exercise modes, initial studies report a mediating effect of exercise mode on event related potentials (P300 amplitude) during task switching [20] and inhibitory control [17] in older adults. In these studies, open skill exercisers exhibited better neural efficiency relative to both closed skill exercisers and a control group.

Taken together, the literature suggests that the effects of a single session of exercise on cognitive performance may be associated with increased arousal [21] and/or, that engaging in physical activity with high cognitive demands maximises the cognitive benefits older adults derive from a single exercise session [22].

These effects of exercise should be particularly evident in perceptual tasks, as arousal alters the signal to noise ratio and increases perceptual sensitivity [23]. Exercise mode could also play a role in exercise induced perceptual benefits, as perceptual adaptation and training can occur rapidly and have an adaptive function [24]. Open skill exercisers are likely more exposed to multisensory stimuli than closed skill exercisers, therefore perceptual learning may be more likely to occur. Perceptual adaptation can be obtained in older individuals [25] and perceptual training can modify audio-visual temporal discrimination [26], even if it naturally deteriorates with ageing [27–29]. These effects are not limited to audio-visual discrimination or integration [30]. Whether exercise modifies perceptual efficiency across different sensory modalities is a relevant question in light of an emerging literature on the functional and cognitive impairments associated with inefficient multisensory processing in ageing [31–33].

Recent studies on temporal discrimination and integration across audition and vision show that older adults present a deficit in such abilities. More specifically, they integrate information across the senses over an excessively large Temporal Binding Window (TBW) [26,27,29,34]. This excessively large TBW in older is associated with negative functional [33,35,36] and cognitive outcomes [32]. More broadly, temporal precision in perceiving external inputs is a fundamental feature of human cognition [37] which has been linked to global cognitive function [38], executive functions [39] and problem solving [40].

There is some initial indication of an association between exercise and cross-sensory integration [31] showing that, in contrast to older adults who do not exercise, those who exercise may need to rely less on multisensory integration to obtain efficient perception. In addition, while research has linked exercise to improved balance [41,42], little has been done to investigate the effect that exercise may have on the cross-modal perceptual processes underlying balance control, which have been associated with maintenance of multisensory perceptual efficiency [35,36].

To date, the few available studies considering perception focus on the visual modality [43,44]. Davranche and colleagues showed that flicker fusion detection is improved by aerobic exercise [43,44]. Thirty minutes of exercise were sufficient to improve performance to the flicker fusion in a 40 minute exercise session and this improvement vanished 30 minutes after exercise cessation [45]. In the same study, no effect was found for a task requiring working memory and inhibition. The authors concluded that perception may be affected by the arousal induced by the exercise (cycling), while higher level cognitive functions were not (although ceiling effects could not be excluded).

This dissociation in effects within the same study speaks to the much debated topic of the specificity of the effect of exercise on cognitive function. Meta-analyses have revealed that physical activity has differential effects on cognition, with some but not all cognitive abilities amenable to exercise-induced benefits [6,16,46]. The most convincing evidence for the positive effects of exercise on cognition has been found in studies of executive function [2,7,16]. Executive functions are arguably the most studied capacity in relation to exercise, because they decline with ageing [47] and are necessary for normal everyday functioning [48,49]. Sub-components of executive function have been studied separately [11,12,46–48]. To date, most research has focused on the inhibitory aspect of executive function [50], which has led to the relative neglect of other subcomponents, including short term memory and, more broadly, working memory [8,16], which is also known to decline with ageing [51]. Short term memory is one of the fundamental and most studied cognitive functions, particularly the Digit Span has been shown to be predictive of higher cognitive function [52], therefore the lack of investigation on its susceptibility to change with exercise is surprising. A recent meta-analytic review addressed the current state of the literature surrounding exercise and memory function focusing on both acute and chronic exercise [and concluded that only chronic exercise had significant positive effects on working memory performance [11]. In the acute exercise literature, inconsistencies can be found, with some reporting a positive effect of acute exercise on working memory [53,54] and others finding no effect [55,56,57]. Interestingly, acute exercise produced stronger effects (small to moderate effects) on working memory compared to chronic exercise [11]. Therefore, it appears from the literature that in the exercise literature two aspects of cognitive function have not been sufficiently studied so far, perception and specifically multisensory perception, and immediate memory.

The present study aimed at assessing the effects of one bout of exercise, either open skill or closed skill, in older adults and compares it with the effects of one session of a sedentary activity. The specific processes tested were multisensory perception and immediate memory. Multisensory perception was assessed by the Sound Induced Flash Illusion (SiFI) [58] and the memory task used was the Forward Digit Span, a working memory task targeting immediate recall ability [59].

The SiFI occurs when two auditory stimuli (e.g. two beeps) are presented in temporal proximity to a single visual stimulus (e.g. a flash) leading to the perception of two visual stimuli (i.e. two flashes). Due to their temporal proximity, the auditory and visual stimuli are integrated, resulting in the incorrect perception of an extra visual stimulus. The SiFI was chosen because it is associated with cross-modal temporal binding windows [60], which are altered by ageing [33]. As we age, the time frame during which proximal information is integrated, becomes extended, leading to an extended temporal window of integration [61]. However this extended temporal window can lead to inaccurate perception and increase the risk of loss of balance [33]. Improvement in balance with a programme of exergame training has also been associated with decreased susceptibility to the illusion [36]. Importantly, measuring susceptibility to this illusion does not require a speeded response, which makes it particularly apt to assess multisensory perception in an older population.

Similar to the Davranche et al. study [43], in addition to a perceptual measure (the SiFI) we also included a measure of memory, in particular immediate memory, as it was noted that this is an understudied, albeit important cognitive capacity that declines with age.

The Forward Digit Span was chosen in light of the lack of studies on immediate memory. Forward Digit Span, which measures the storage aspect of working memory function, is one measure frequently included in neuro-cognitive batteries [62,63]. While digit span features regularly in studies of chronic exercise [46,49,64,65], it is rarely included in studies with an acute exercise paradigm [11]. Two existing studies examined young and middle-aged adults [56,66]. A study on 58 middle aged adults found no effect of 40 minutes of walking on digit span performance [56]. In contrast, Davey and colleagues found improved digit span performance following just 30 seconds of cycling in an undergraduate sample [66]. To our knowledge, there is no existing study investigating the effect of acute exercise on digit span performance in an older population.

Based on existing research in this field, it is predicted that a single session of exercise will facilitate cognitive and perceptual functioning, resulting in more efficient multisensory integration and improved immediate memory in the exercise groups in comparison with the sedentary activity control group (Hypothesis 1). Furthermore, it is expected that exercise modality will moderate this effect of exercise, such that a single session of open skill exercise will lead to greater benefits compared to a single session of closed skill exercise (Hypothesis 2). The relationship between chronic exercise habits and the effects of one bout of exercise on cognition will be also investigated, with the hypothesis that more frequent habitual exercise will be associated with greater cognitive benefits following the session of exercise (Hypothesis 3).

## Method

### Participants

58 older adults (37 female) were recruited for participation in this study. Participant age ranged from 60 to 81 (*M* = 69.65, *SD* = 5.64). Participants fell into one of three groups: open skill exercise, closed skill exercise and a control group. Exercise participants were recruited from local gyms and fitness centres (*n* = 37). Control participants were recruited from active retired groups (*n* = 21). Exercise participants were subdivided into two categories; open skill exercisers (*n* = 18) and closed skill exercisers (*n* = 19). This categorisation was based on the type of exercise participants performed during the experimental period as their normal exercise routine. Participants in the open skill group engaged in aerobics class, tennis or dance classes. Closed skill participants engaged in exercise such as swimming or gym circuits. The control group engaged in a sedentary activity such as an active retired group meeting or card games. Participants in this group were also tested before and after one session of their sedentary activity in order to be able to compare the effect of a cognitively stimulating activity with no exercise component. No activity, either exercise or sedentary, was experimentally induced as all activities were part of participants’ usual routine. Each open skill exercise session lasted approximately 80 ± 20 minutes (including a short break), the closed skill session lasted 70 ± 20 minutes (including a short break) and the control session had a 60 minute duration.

All participants had normal or corrected-to-normal vision and hearing by self-report. All participants were screened for cognitive impairment using the Quick Mild Cognitive Impairment Screen (Q-mci) [67], and did not meet the criterion for Mild Cognitive Impairment [68]. The Q-mci test was adopted because it appears particularly sensitive to mild cognitive impairment and therefore useful for the healthy population included here. None of the participants reported a diagnosis of a neurological or psychiatric condition (i.e. Parkinson’s, dementia, Alzheimer’s, depression or anxiety). Additional health conditions assessed by self-report included the following cardiac conditions; high blood pressure, heart attack, stroke, mini-stroke, high cholesterol or diabetes, as well as substance or alcohol abuse. Participants were also assessed for their physical activity level by the International Physical Activity Questionnaire Short Form (IPAQ-SF) [69]. The IPAQ-SF is validated for use on adults aged 18–65 [69]. Validity is not established for older adults but the questionnaire has been used in recent studies on older adults [70,71]. Table 1 reports the characteristics of the sample. The groups did not significantly differ in age, education, Q-mci, self-reported vision, or health. The control group reported better day-to-day memory compared to the closed skill group and better hearing compared to both the open and closed skill group. As expected, the groups also differed in their physical activity levels (MET-minutes).

**Table 1:** Participants who completed digit span task at Time 1 and Time 2.

	Open Skill	Closed Skill	Control	p value^
*N*	18	19	21	
Age (in years)*	69.22 (5.09)	69.16 (4.8)	70.48 (6.86)	0.7
Sex	Male 5.6%, Female 94.4%	Male 63.2%, Female 36.8%	Male 38.1%, Female 61.9%	0.001 (Open skill vs. control)
IPAQ*	3997.24 (2313.9)	2926.89 (1702.25)	1375 (1325.89)	0.001 (Open skill vs. control, closed skill vs. control)
Education	Primary 5.6%, Secondary 38.9%,	Primary 10.5%, Secondary 47.4%,	Primary 9.5%, Secondary 47.6%,	0.53
	3rd level 50%, Postgrad 5.6%	3rd level 26.3%, Postgrad 15.8%	3rd level 42.9%, Postgrad 0%	
Physical Health	Ex 50%, Vg 38.9%, G 11.1%	Ex 36.8%, Vg 26.3%, G 31.6%, F 5.3%	Ex 52.4%, Vg 38.1%, G 9.5%	0.41
Mental Health	Ex 50%, Vg 38.9%, G 11.1%	Ex 36.8%, Vg 42.1%, G 21.2%	Ex 71.4%, Vg 28.6%	0.12
Hearing	Ex 5.6%, Vg 50%, G 38.9%, F 5.6%	Ex 15.8%, Vg 36.8%, G 42.1%, F 5.3%	Ex 42.9%, Vg 47.6%, G 9.5%	0.03 (Control vs. open skill, control vs. closed skill)
Eyesight	Ex 33.3%, Vg 55.6%, G 11.1%	Ex 31.6%, Vg 42.1%, G 21.1%, F 5.3%	Ex 38.1%, Vg 52.4%, G 9.5%	0.85
Memory	Ex 5.6%, Vg 72.2%, G 22.2%	Ex 10.5%, Vg 31.6%, G 21.1%, F 15.8%	Ex 42.9%, Vg 33.3%, G 23.8%	0.005 (Control vs. closed skill)
Cardiac Condition	Yes 5.6%, No 94.4%	Yes 26.3%, No 73.7%	Yes 14.3%, No 85.7%	0.33
Other Condition	None 74.4%, Psychiatric 5.6%	None 100%	None 95.2%, Psychiatric 4.8%	0.63
Depressed	Rarely 100%	Rarely 94.7%, Sometimes 5.3%	Rarely 100%	0.64
Q-mci*	74.83 (4.48)	71.53 (8.26)	70.24 (7.18)	0.11

* denotes scores expressed as mean with standard deviation in parentheses.

^ P values refer to analyses including the 3 groups, specified by post hoc tests or planned comparisons. Chi-square tests were used for categorical variables (Fisher’s exact test was used in instances where the assumption of expected frequency was not met).

T1, time 1; T2, time 2; Ex, excellent; Vg, very good; G, good; F, fair; P, poor; IPAQ, international physical activity questionnaire; Q-mci, Quick Mild Cognitive Impairment Screen.

All participants provided written informed consent prior to participation. This study was approved by the School of Applied Psychology Ethics Committee, University College Cork.

### Material

For the SiFI [58], the visual stimulus consisted of a white disk of radius 1.5° (flash) presented on a black background screen for a duration of 16ms, 5cm below fixation. The auditory stimulus consisted of a beep (3500 Hz) presented for 10ms (1ms ramp).

The forward digit span is a modified version of a subtest (Forward Digit Span) of the WAIS-IV [59].

### Procedure

Participants were required to complete two testing sessions. At the first session (T1), participants were briefed on the nature of the study, asked to provide written informed consent and screened for cognitive impairment using the Q-mci. Participants then completed a series of interviewer administered questionnaires; a health and demographic questionnaire, the IPAQ-SF and the L-PAQ. All questionnaire items were read aloud to the participants and their verbal responses were written down by the experimenter. Next, participants completed baseline tests of cognition and perception, namely the Forward Digit Span task and the SiFI task. All participants completed the Digit Span task first followed by the SiFI.

For the Digit Span, digits were presented at a rate of one digit per second. All digit series were read in an even tone by the experimenter. There were a total of 14 trials. Trials were presented in blocks of two, with each block containing two trials of equal complexity (i.e. same number of digits presented in both trials). After successfully completing one block, participants were presented with the next block. Each successive block contained an additional digit and hence increased in complexity. The Digit Span task was discontinued once a participant either successfully completed all 14 trials or incorrectly repeated two trials of the same block (i.e. two digit series of the same length).

For the SiFI, participants sat in front of the computer screen at approximately 50cm and were asked to look at the fixation cross and count the white dots (flashes) flashing rapidly on the screen. The flashes could be presented with 2 beeps, which participants were asked to ignore. The experiment comprised illusory conditions with variable Stimulus Onset Asynchrony (SOA) of 70, 150 and 230ms (5 repetitions), control conditions where 1 flash was presented with 1 beep (10 repetitions), and 2 flashes were presented with 2 beeps (with the same SOAs as for the illusory conditions) and 2 flashes only (with the same SOAs as for the illusory conditions) (5 repetitions each). The total number of trials was 55, with conditions presented in random order for each participant. Participants also completed a separate block in which they were asked to report how many beeps they heard (either 1 beep or 2 beeps presented with the different SOAs). This block comprised of 10 ‘1 beep’ trials and 30 ‘2 beeps’ trials (10 at each of the three SOAs). The beeps only block was always the second block.

The SiFI task was administered using a Toshiba Satellite Laptop (screen dimensions: 34 × 19cm, model number: PSKINE-01500MEN) and Windows 7 processor. The SiFI task was programmed and run using either the E-Prime 2.0 software suite (Psychology Software Tools, 2007) or Presentation software (www.neurobs.com, version 18.1).

The second experimental session (T2) occurred within 10 minutes of participants completing a session of exercise (i.e. open skill or closed skill exercise) or a sedentary activity in the case of the control group. This post-test session involved completing the Forward Digit Span task and the SiFI task for a second time. The experimental protocol lasted 35 minutes in total (excluding the exercise session); the first session had a duration of approximately 25 minutes and the second session lasted 10 minutes.

## Results

### Digit span

Performance on the Forward Digit Span was calculated as the digit span score (corresponding to the block where the participants reported correctly to at least one of the two strings of numbers, minimum = 0, maximum = 8) and the digit span product score (corresponding to the digit span score by the number of correct strings reported). The product score constitutes a way to give higher weight to consistently correct performance, as participants who have more often reported correctly both lists per block obtain a higher product score (see Table 1). A two way ANOVA was conducted on digit span scores, with Time (Time 1, Time 2) as the within subjects factor and Group (open skill, closed skill, control) as the between subjects factor. The ANOVA revealed no significant main effect of Group [*F*(2, 55) = .30, *p* = .74, partial eta squared = .01]. The main effect of Time [*F*(1, 55) = 3.53, *p* = .06, partial eta squared = .06] and the Time × Group interaction effect [*F*(2,55) = 2.64, *p* = .05, partial eta squared = .08] just failed to reach significance (see S1 Fig).

**Fig 1:**
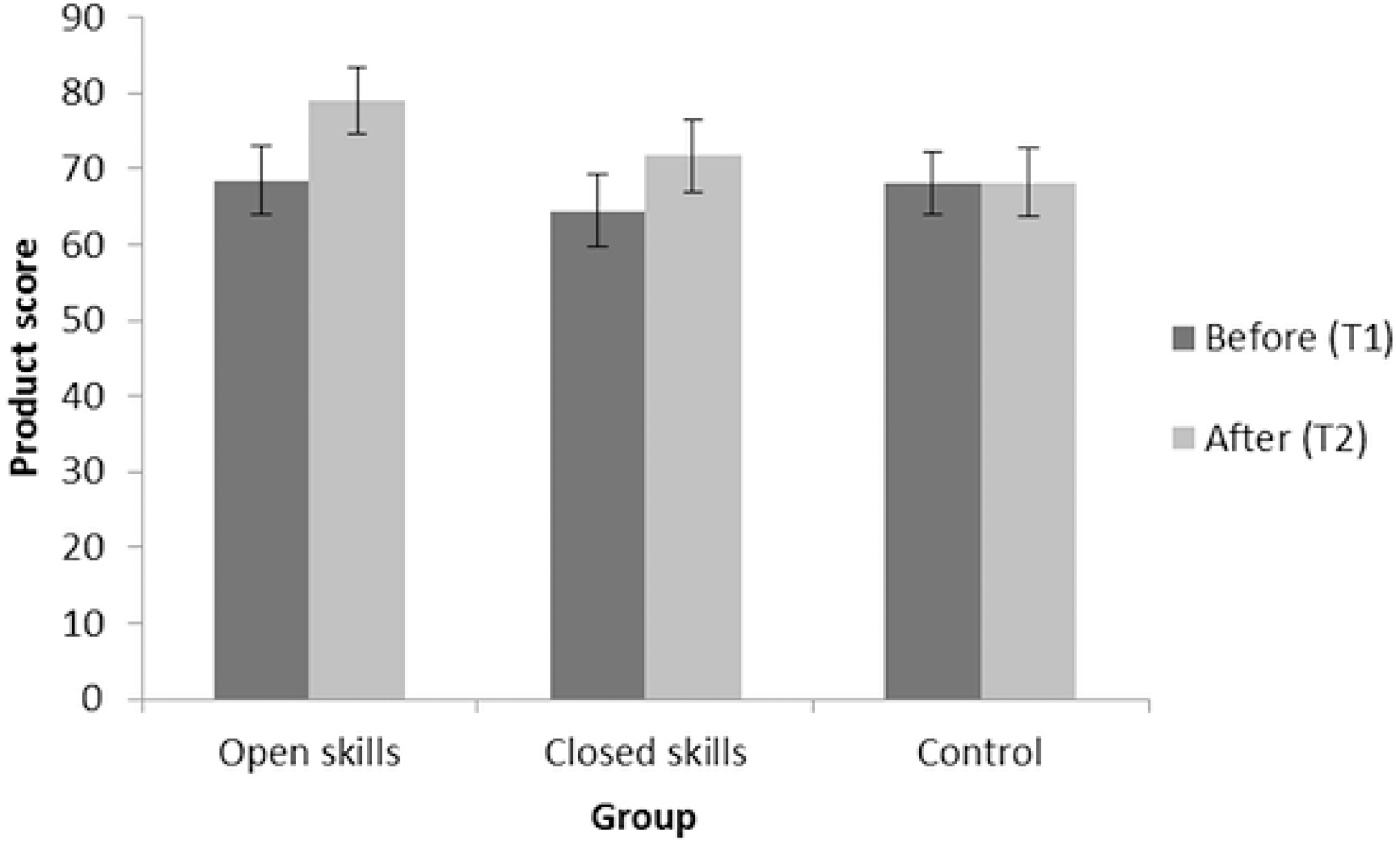
Digit span product scores for the three groups of participants before and after their activity. T1, time 1; T2, time 2.

The same mixed measures two way ANOVA on the product score showed a main effect of Time [*F*(1, 55) = 13.06, *p* = .001, partial eta squared = .19], a significant Time × Group interaction [*F* (2, 55) = 3.57, *p* < .05, partial eta squared = .12], and no main effect of Group [*F*(2, 55) = .56, *p* = .58, partial eta squared = .02]. Refer to Fig 1 for the Time × Group interaction. Planned paired samples t-tests revealed the open skill group significantly improved from T1 to T2 (*t*(17) = 2.87, *p* < 0.01, *d* = 0.68), as did the closed skill group (*t*(18) = 2.18, *p* < 0.05, *d* = 0.5), while no difference was found for the control group (*t*(20) = .72, *p* = 0.94, *d* = 0.02). However when Holm-Bonferroni corrected, only the open skill group significantly improved from T1 to T2 (*p* < .05), while only a trend was found for the closed skill (*p* = .086). To explore whether the open and closed skill groups differed in the magnitude of improvements post-exercise, a series of Bonferroni-corrected independent samples t-tests were conducted between groups. No significant difference was found between open and closed skill groups on post-exercise (T2) product scores (*t*(35) = 1.13, *p* = .27, *d* = 0.38). This indicates that while the open skill group seem to more robustly improve post exercise; this improvement was not sufficient to obtain significant difference post exercise (no difference between the groups pre-exercise was present). An additional analysis using random permutations was conducted to confirm the robustness of the interaction given the relatively small sample. Permutations were restricted to occur within the factor group and the assignment of participants to the other factor (i.e. Digit Span Product) was kept constant. When testing the main effect of Group, the factor Digit Span Product is held constant. The analysis confirmed the Time × Group interaction as significant [*F* = 3.5742, *p* = 0.039] also suggesting the more robust improvement at T2 for the open skill group.

**Fig 2:**
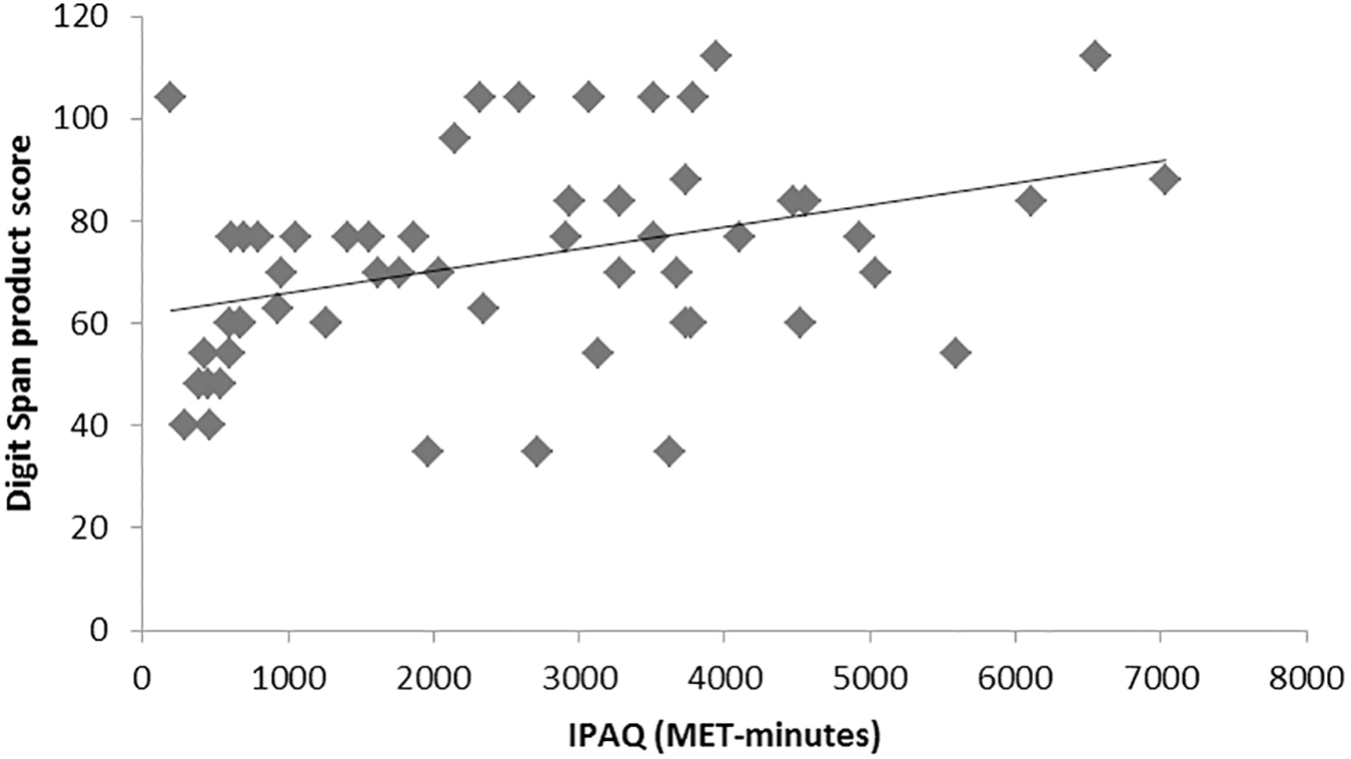
Correlation between digit span product score at Time 2 and exercise in the previous week. IPAQ, international physical activity questionnaire; METs, metabolic equivalents.

Due to the quasi-experimental nature of the design, two factors related to the physical activity conducted by participants overlay in the study: the distinction between open skill, closed skill and control and the amount of habitual exercise for participants in each group, as shown by the significant difference in MET-minutes in the IPAQ (see Table 1 and S1 Table). To explore the association between IPAQ scores and belonging to a given group in relation to the Digit Span performance, correlations between the MET-minutes and the Digit Span product score were conducted. The correlation between the score at T1 and the MET-minutes was not significant [*r*^*2*^ = 0.02, *p* = 0.31], while the correlation between score at T2 and MET-minutes was significant [*r*^*2*^ = 0.12, *p* = 0.01]. This correlation remained significant even after removing an outlier with extremely high MET-minutes (i.e. 11278) (see Fig 2). To explore this correlation further, a multiple regression was conducted with Digit Span product score at T2 as the outcome variable and the predictor factors being IPAQ (MET-minutes), age (typically associated with span performance), baseline product score (T1) and Group. The analysis revealed a significant effect of the baseline product score and of the IPAQ, but no significant effect of Group (the Beta Coefficients are available in S2 Table). This indicates that the habitual amount of exercise was associated with memory benefits more than the mode of exercise bout per se.

### Sound-induced flash illusion

Eight participants did not perform the SiFI due to their personal time constraints; therefore the sample for the analyses reported below is 50 participants. Their characteristics do not differ from the overall sample and are reported in S1 Table. The proportion of correct responses for each of the illusion, control and unisensory conditions was calculated. Mixed model analyses of variance were used to assess differences in proportion of correct responses. Greenhouse-Geisser correction was applied when there was lack of homogeneity of variance.

Due to the presence of a baseline group difference in self-reported hearing, an analysis was conducted to ensure no baseline group differences in participants’ ability to hear the auditory stimuli. A one way ANOVA was conducted on participant responses to beep only trials (i.e. averaged accuracy to one beep and two beep trials), with Group as a factor. There was no main effect of group on accuracy [*F*(2, 47) = .84, *p* = .44], indicating that groups do not differ in their ability to discriminate the auditory experimental stimuli.

**Fig 3:**
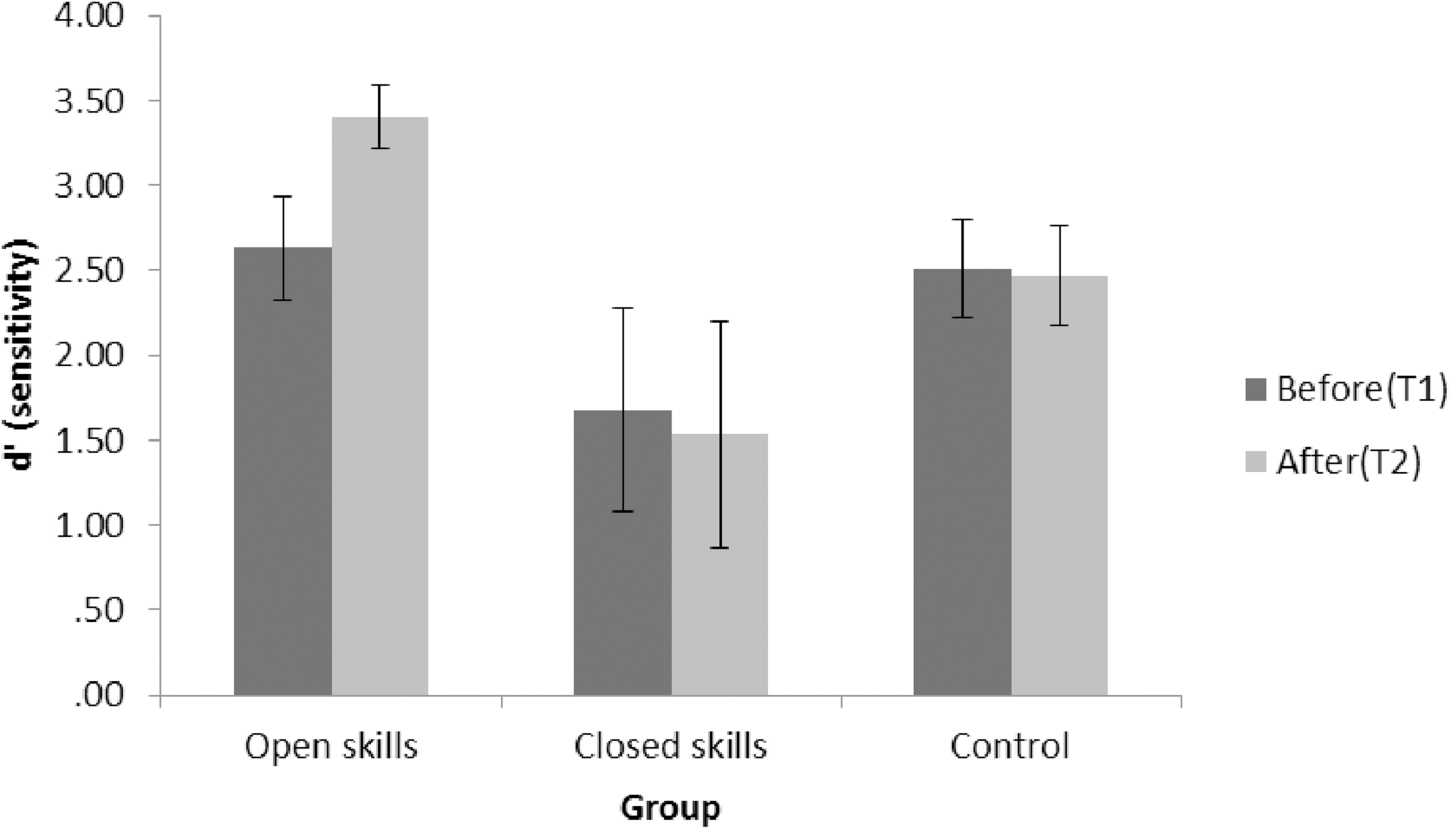
Sensitivity in detecting 2 real flashes when presented with 2 beeps as opposed to detecting 2 illusory flashes as false alarm. T1, time 1; T2, time 2; d’, d prime.

To assess whether groups had underlying differences in perceiving congruent conditions, a three way ANOVA: 3 (group: open skill, closed skill, control) × 3 (SOA: 70ms, 150ms, 230ms) × 2 (Time: Time 1, Time 2) was conducted on congruent trials (2 flashes / 2 beeps). Results revealed a main effect of Group [*F*(2, 47) = 5.43, *p* < .01, partial eta squared = .18], no other main effect or interaction was significant. Post hoc comparisons showed this effect was driven by both the open skill (*p* < .05) and control group (*p* < .05) performing significantly better on congruent trials compared to the closed skill group (mean = 0.94; st. dev. = .038; mean = 0.98, st. dev. = 0.039; mean = 0.80, st. dev. = 0.038 respectively). The same 3 x 3 x 2 ANOVA was utilised to analyse the proportion of correct responses to unisensory trials (2 flashes). The results also indicated a main effect of Group [*F*(2, 47) = 3.82, *p* < .05, partial eta squared = .14], whereby the proportion of correct responses for the control group was higher than for the closed skill group [*p* <0.05, mean = 0.97, st. dev. = 0.037; mean = 0.80, st. dev. = 0.038 respectively]. A main effect of Time [*F*(2, 47) = 5.79, *p* < .05, partial eta squared = .11], indicated that participants were more correct in reporting 2 flashes after exercise (mean = 0.98, st. dev. = 0.02) than before (mean = 0.88, st. dev. = 0.02). Consequently, a sensitivity analysis procedure (i.e. d’ analysis) was conducted to control for these baseline group differences. Signal Detection Theory was used to detect sensitivity changes (*d*’) in correctly perceiving two flashes with two beeps (hits) compared to perceiving two flashes in the illusion conditions (i.e. false alarms). D prime scores (*d′*) were calculated as [*z*(hits)–*z*(false alarms)] [72]. In the formula, *z* represents the inverse cumulative normal. The *d′* was calculated for each SOA separately. Normality tests (Kolmogorov-Smirnov) revealed that the data for all *d′* dependent variables was normally distributed (average *d′* scores for T1, average *d′* scores for T2, the overall *d′* scores across T1 and T2). A 3 way ANOVA was conducted on *d′* scores with Group, Time and SOA as factors. There was a significant main effect of SOA [*F*(2, 96) = 7.16, *p* = .001, partial eta squared = .13]. Post hoc (Tukey) on the main effect of SOA found significantly lower sensitivity at the shortest SOA (70ms) compared to the longer SOAs (150ms and 230ms) (*p* < .001). The main effect of Group just failed to reach significance [*F*(2, 47) = 2.99, *p* = .059, partial eta squared = .11]. The interaction effect between Time and Group was significant [*F*(2, 47) = 4.54, *p* < 0.05, partial eta squared = .16]. No other interaction was significant. Planned comparisons (Holm-Bonferroni corrected paired samples t-tests) revealed that the open skill group significantly improved on *d’* scores from Time 1 to Time 2 (*t*(16) = .3, *p* = .027, *d* = .77), while the closed skill and control group performance did not change significantly from Time 1 to Time 2 (see Fig 3). To test for between-groups differences in post-exercise (T2) scores, post hoc tests (using Holm-Bonferroni corrected independent samples t-tests) were conducted. This analysis revealed that at T2, the open skill group performed significantly better on *d’* scores compared to both the closed skill [*t*(18.5) = 2.7, *p* = .033, *d* = 0.93] and the control group [*t*(31) = 2.72, *p* = .033, *d* = 0.95]. The closed skill and control group did not significantly differ in *d’* scores at T2 [*t*(21.93) = 1.28, *p* = .21, *d* = 0.45]. An inspection of the means shows that the closed skill group had lower sensitivity than the other groups. To investigate this difference further, a 3 way ANCOVA was conducted with the global cognition score (Q-mci) and age as covariates. This analysis only revealed a significant interaction between Group and Time [*F*(2, 45) = 3.23, *p* < 0.05, partial eta squared = .13] due to the open skill group being the only group to show improvement between T1 and T2 (*p* <0.01).

Analysis on the *d*’ was also conducted with random permutation ANOVA showing a main effect of Group [*F* = 2.99, *p* = 0.048] and the Time by Group interaction [*F* = 4.54, *p* = 0.01].

To explore the relationship between overall quantity of physical activity and susceptibility to the SiFI, correlations were conducted between the *d’* score (averaged across SOAs) at T1 and T2 with the IPAQ score (MET-minutes), revealing no significant correlation [*d’* at T1 and IPAQ: *r*^*2*^ = 0.006, *p* = 0.59; *d’* at T1 and IPAQ: *r*^*2*^ = 0.06, *p* = 0.09]. In addition, a multiple regression with the outcome variable being the *d’* score at T2 and predictors MET-minutes, Group, Age and *d’* score at T1 revealed only a significant effect for the *d’* score at T1 and for Group (beta coefficients available in S3 Table).

## Discussion

This study aimed at assessing whether multisensory perception and immediate memory could be improved by a single bout of exercise. Three groups of older adults were tested before and after one session of their usual activity, which could be either open skill, closed skill or sedentary. The results showed that both open and closed skill exercisers had more efficient immediate memory after their exercise session, while the control participants who conducted a sedentary activity did not show such improvements. Improvements were evident in the product score of the Digit Span, indicating that exercisers provided a more consistent performance in recollecting the items than the control group. The effect at T2 was correlated with MET-minutes in the previous week and regression analysis found the level of habitual physical activity was the best predictor of performance at T2, possibly indicating that this is a moderator for the beneficial effects of one exercise bout on immediate memory. This suggests that the positive benefits of exercise on memory are favoured by sustained exercise regime, while the exercise mode was not relevant: open skill exercisers were habitually more active than closed skill exercisers and this was reflected in the more robust effect for the first group. This finding is in line with those reported by Hopkins and colleagues [8]. A more detailed analysis of habitual exercise modes, which was not possible here, may allow further insight into the benefits of one bout of exercise.

Differently, the positive effect of exercise on cross-modal perception efficiency were only evident for the open skill group, while no sensitivity benefit was found for the closed skill and control group. The level of physical activity in the previous week (MET-minutes) was neither correlated with nor a predictor of scores at T2, indicating that habitual physical activity is not the main factor in perceptual improvements. This is in line with the hypothesis that this effect is due to the temporary arousal and associated with increased signal to noise ratio. This is also consistent with previous findings showing increased sensitivity to the flicker fusion effect after 30 minutes of cycling [45]. However, the present results differ from these previous findings in that the only activity giving rise to significant positive effects in the present study was open skill exercise. Considering that exercise intensity was not measured here, we cannot ascertain whether the results are due to a quantitative difference in the intensity of the exercises or a qualitative difference between the two exercise modes. However, it is useful to note that the open skill exercisers in this study were exposed to a multisensory environment, while on the contrary, closed skill exercisers were conducting activities affording concentration on self more than the environmental stimulation, such as swimming. While participants in the control group were exposed to some multisensory stimulation (e.g. having a group conversation), this is likely qualitatively different from open skill exercise, where cross-modal stimulation was essential to the exercise itself and required a full-body engagement (i.e. dancing or aerobic-dance). These differences need to be explored further.

The main limitations of this study are due to the quasi-experimental design. First, participants were not randomly assigned to groups, but selected based on their current activities. However, the baseline measures of health and cognitive status indicate that no significant differences existed between these groups of healthy older adults. While a number of health conditions did not differ across groups, including cardiac, chronic, psychiatric and memory-related conditions, future studies should consider controlling for a wider list of potential comorbidities influencing exercise habits, for example orthopaedic issues and body mass index. A second notable limitation is the relatively small sample size, which precluded an analysis on potential gender effects. Finally, the kinds of activities were not under the experimenter’s control, therefore a certain variety within each category was contemplated. Nonetheless, there is a clear distinction between the activities in which the three groups of participants were involved. Pertinently, the dissociation between the findings for immediate memory and for cross-modal perception suggests that different dimensions of the session of activity may influence differently participants’ capabilities. The different results between the open skill and control group also allow us to suggest that it is not social interaction which drives the open skill groups improved performance, as the control group activity also included a social component.

In conclusion the first hypothesis on the benefits of exercise for cross-modal perception and immediate memory was verified. The second hypothesis, on exercise mode, was verified for cross-modal perception, while no difference between open and closed skill exercise was found for immediate memory. Instead habitual exercise was predictive of memory performance post exercise. While the confounding factor of exercise intensity should be emphasised, these findings offer support to the proposal that, globally, open skill exercise is more apt to produce short term perceptual and cognitive benefits than closed skill exercise. The third hypothesis on the role of exercise habits was only verified for immediate memory, and not for cross-modal perception.

Considering the small sample size and quasi-experimental paradigm, these findings should be considered a first step in elucidating the role of one bout of exercise on multisensory perception and immediate memory, two neglected cognitive skills in the exercise literature, with further research needed with larger samples and a randomised controlled trial paradigm.

The present study, despite being limited in determining the causal pathways for the effects found, constitutes the first example to our knowledge of modulation of cross-modal perception by one session of exercise in older adults. This work also shows a dissociation in the effects of exercise mode on perception and cognition, with both exercise modes providing an advantage to immediate memory, while only open skill exercise showing positive effects on cross-modal perception. To understand whether this is related to arousal, or a more specific effect of ‘perceptual training’ provided particularly by open skill exercise remains to be investigated further. Clearly, the present results show that exercise appears to be a pathway to counteract decline of cross-modal perception in ageing and its function and cognitive consequences, as well as being a viable way to sustain immediate memory which has notable potential applied implications.

## Supporting information

S1 Fig Digit span scores for the three groups of participants before and after their activity. T1, time 1; T2, time 2.
(DOCX)

S1 Table Participants who completed perception experiment at Time 1 and Time 2. (DOCX)

S2 Table Beta coefficients from multiple regression analysis on digit span product scores at Time 2. (DOCX)

S3 Table Beta coefficients from multiple regression analysis on *d*’ prime scores at Time 2. (DOCX)

S1 Dataset (XLSX)
