# Supplementary material for: One bout of open skill exercise improves cross-modal perception and immediate memory in healthy older adults who habitually exercise
Source: PLoS One. 2017 Jun 1;12(6):e0178739. doi: 10.1371/journal.pone.0178739 (PMC5453579; doi:10.1371/journal.pone.0178739)
Supplement: S1 Fig — T1, time 1; T2, time 2. (DOCX) [file pone.0178739.s001.docx]

**S1 Fig. Digit Span product scores for the three groups of participants before and after their activity.** T1, time 1; T2, time 2. The Time × Group interaction approached significance (*p* = .05).
