# Supplementary material for: One bout of open skill exercise improves cross-modal perception and immediate memory in healthy older adults who habitually exercise
Source: PLoS One. 2017 Jun 1;12(6):e0178739. doi: 10.1371/journal.pone.0178739 (PMC5453579; doi:10.1371/journal.pone.0178739)
Supplement: S1 Table — (DOCX) [file pone.0178739.s002.docx]

| **S1 Table. Participants who completed perception experiment at Time 1 and Time 2.** | | | | |
| --- | --- | --- | --- | --- |
|  |  |  |  | |
|  | **Open Skill** | **Closed Skill** | **Control** | |
| *N* | 17 | 17 | 16 | |
| Age (years)* | 68.59 (4.46) | 69.59 (4.82) | 68.69 (6.71) | |
| Sex | Female 94.1%, Male 5.9% | Female 35.3%, Male 64.7% | Female 62.5%, Male 37.5% | |
| Education | Primary 5.9%, Secondary 41.2%, 3rd level 47.1% | Primary 11.8%, Secondary 41.2%, 3rd level 29.4% | Primary 0%, Secondary 43.6%, 3rd level 58.2% | |
|  | Postgrad 5.9% | Postgrad 17.6% | Postgrad 0% | |
| Physical Health | Ex 52.9%, Vg 35.3%, G 11.8% | Ex 41.2%, Vg 29.4%, G 29.4% | Ex 62.5%, 31.2%, 6.2% | |
| Mental Health | Ex 52.9%, Vg 35.3%, G 11.8% | Ex 29.4%, Vg 47.1%, G 23.5% | Ex 75%, Vg 25% | |
| Hearing | Ex 5.9%, Vg 52.9%, G 41.2% | Ex 17.6%, Vg 35.3%, G 41.2%, F 5.9% | Ex 50%, Vg 37.5%, G 12.5% | |
| Eyesight | Ex 35.3%, Vg 58.8%, G 5.9% | Ex 35.3%, Vg 35.3%, G 23.5%, F 5.9% | Ex 50%, Vg 37.5%, G 12.5% | |
| Memory | Ex 5.9%, Vg 70.6%, G 23.5% | Ex 11.8%, Vg 35.3%, G 35.3%, Fair 17.6% | Ex 31.2%, Vg 37.5%, G 31.2% | |
| Cardiac Condition | Yes 5.9%, No 94% | Yes 23.5%, No 76.5% | Yes 6.2%, No 93.8% | |
| Other Condition | None 94.1%, Psychiatric 5.9% | None 100% | None 100% | |
| Depressed | Rarely 100% | Rarely 94.1%, Sometimes 5.9% | Rarely 100% | |
| IPAQ score (in METS min/week)* | 4170.82 (2262.12) | 3127.06 (1678.69) | 1528.13 (1448.52) | |
| LPAQ Past Year (METs hours/week)* | 25.97 (12.16) | 23.28 (9.67) | 17.1 (6.75) | |
| LPAQ Total (METs hours/week)* | 32.17 (12.52) | 33.47 (14.76) | 24.77 (14.7) | |
| IPAQ category | sufficient 76.5%, low 23.5% | sufficient 41.2%, low 52.9%, inactivity 5.9% | sufficient 31.2%, low 37.5%, inactivity 31.2% | |
| Length of Digit Span T1* | 6.59 (.87) | 6.65 (1) | 6.69 (.79) | |
| Length of Digit Span T2* | 7.06 (.83) | 6.76 (.83) | 6.69 (.87) | |
| Qmci | 75.41 (3.86) | 71.24 (8.64) | 70.81 (7.92) | |
| *Note. ** denotes scores expressed as mean with standard deviation in parentheses. Ex, excellent; VG, very good; G, good; F, fair; P, poor; T1, time 1; T2, time 2; MET, metabolic equivalent. | | | |  |
