# Supplementary material for: One bout of open skill exercise improves cross-modal perception and immediate memory in healthy older adults who habitually exercise
Source: PLoS One. 2017 Jun 1;12(6):e0178739. doi: 10.1371/journal.pone.0178739 (PMC5453579; doi:10.1371/journal.pone.0178739)
Supplement: S2 Table — (DOCX) [file pone.0178739.s003.docx]

**S2 Table. Beta coefficients from multiple regression analysis on digit span product scores at Time 2.**

|  | **DSproductafter Param.** | **DSproductafter Std.Err** | **DSproductafter t** | **DSproductafter p** | **-95.00% Cnf.Lmt** | **+95.00% Cnf.Lmt** | **DSproductafter Beta (ß)** | **DSproductafter St.Err.ß** | **-95.00% Cnf.Lmt** | **+95.00% Cnf.Lmt** |
| --- | --- | --- | --- | --- | --- | --- | --- | --- | --- | --- |
| **Intercept** | -28.997 | 31.592 | -0.918 | 0.363 | -92.392 | 34.398 |  |  |  |  |
| **Age** | -0.145 | 0.339 | -0.430 | 0.669 | -0.825 | 0.534 | -0.041 | 0.095 | -0.231 | 0.150 |
| **IPAQ** | 0.002 | 0.001 | 2.246 | 0.029 | 0.000 | 0.004 | **0.239** | 0.106 | 0.026 | 0.453 |
| **DSLengthBefore** | 15.925 | 2.188 | 7.278 | 0.000 | 11.534 | 20.315 | **0.674** | 0.093 | 0.488 | 0.860 |
| **Open skill** | 3.538 | 2.831 | 1.250 | 0.217 | -2.143 | 9.219 | 0.145 | 0.116 | -0.088 | 0.378 |
| **Closed skills** | -1.583 | 2.512 | -0.630 | 0.531 | -6.625 | 3.458 | -0.066 | 0.104 | -0.276 | 0.144 |

*Note.* IPAQ, International Physical Activity Questionnaire; T1, Time 1; T2, Time 2.
