# Supplementary material for: One bout of open skill exercise improves cross-modal perception and immediate memory in healthy older adults who habitually exercise
Source: PLoS One. 2017 Jun 1;12(6):e0178739. doi: 10.1371/journal.pone.0178739 (PMC5453579; doi:10.1371/journal.pone.0178739)
Supplement: S3 Table — (DOCX) [file pone.0178739.s004.docx]

**S3 Table**. **Beta coefficients from multiple regression analysis on *d’* scores at Time 2.**

|  | **AveragedprimeAfter Param.** | **AveragedprimeAfter Std.Err** | **AveragedprimeAfter t** | **AveragedprimeAfter p** | **-95.00% Cnf.Lmt** | **+95.00% Cnf.Lmt** | **AveragedprimeAfter Beta (ß)** | **AveragedprimeAfter St.Err.ß** | **-95.00% Cnf.Lmt** | **+95.00% Cnf.Lmt** |
| --- | --- | --- | --- | --- | --- | --- | --- | --- | --- | --- |
| **Intercept** | 0.795 | 1.852 | 0.429 | 0.670 | -2.938 | 4.528 |  |  |  |  |
| **Age** | -0.009 | 0.026 | -0.353 | 0.726 | -0.062 | 0.043 | -0.025 | 0.072 | -0.170 | 0.119 |
| **IPAQ** | 0.000 | 0.000 | 1.499 | 0.141 | 0.000 | 0.000 | 0.126 | 0.084 | -0.043 | 0.295 |
| **AveragedprimeBefore** | 0.866 | 0.080 | 10.846 | 0.000 | 0.705 | 1.027 | 0.792 | 0.073 | 0.645 | 0.939 |
| **Open skill** | 0.479 | 0.211 | 2.275 | 0.028 | 0.055 | 0.904 | **0.205** | 0.090 | 0.023 | 0.387 |
| **Closed skill** | -0.431 | 0.196 | -2.201 | 0.033 | -0.826 | -0.036 | **-0.185** | 0.084 | -0.354 | -0.016 |

*Note.* IPAQ, International Physical Activity Questionnaire; T1, Time 1; T2, Time 2.
